# Metallothionein 3 Inhibits 3T3-L1 Adipocyte Differentiation via Reduction of Reactive Oxygen Species

**DOI:** 10.3390/antiox12030640

**Published:** 2023-03-04

**Authors:** Yuankuan Li, Sung Ho Lee, Meiyu Piao, Hyung Sik Kim, Kwang Youl Lee

**Affiliations:** 1 College of Pharmacy, Research Institute of Pharmaceutical Sciences, Chonnam National University, Gwangju 61186, Republic of Korea; lyk208943@jnu.ac.kr (Y.L.); puzim23@jnu.ac.kr (S.H.L.); 186065@jnu.ac.kr (M.P.); 2 School of Pharmacy, Sungkyunkwan University, 2066, Seobu-ro, Jangan-gu, Suwon 16419, Republic of Korea

**Keywords:** metallothionein 3, adipocyte differentiation, reactive oxygen species, PPARγ

## Abstract

Metallothionein 3 (MT3), also known as a neuronal growth-inhibitory factor, is a member of the metallothionein family and is involved in a variety of biological functions, including protection against metal toxicity and reactive oxygen species (ROS). However, less is known about the role of MT3 in the differentiation of 3T3-L1 cells into adipocytes. In this study, we observed that MT3 levels were downregulated during 3T3-L1 adipocyte differentiation. *Mt3* overexpression inhibited adipocyte differentiation and reduced the levels of the adipogenic transcription factors C/EBPα and PPARγ. Further analyses showed that MT3 also suppressed the transcriptional activity of PPARγ, and this effect was not mediated by a direct interaction between MT3 with PPARγ. In addition, *Mt3* overexpression resulted in a decrease in ROS levels during early adipocyte differentiation, while treatment with antimycin A, which induces ROS generation, restored the ROS levels. *Mt3* knockdown, on the other hand, elevated ROS levels, which were suppressed upon treatment with the antioxidant N-acetylcysteine. Our findings indicate a previously unknown role of MT3 in the differentiation of 3T3-L1 cells into adipocytes and provide a potential novel target that might facilitate obesity treatment.

## 1. Introduction

Obesity has been gradually turning into a global epidemic with an increasing prevalence among adults and children [1,2,3]. By 2025, the incidence of obesity is predicted to be more than 21% in women and 18% in men [4]. Obesity is associated with an increased risk of developing various diseases, including several types of cancer [5,6,7], type 2 diabetes [8], and cardiovascular disease [9], which are the primary causes of death worldwide. Therefore, it is crucial to develop effective interventions for the prevention and treatment of obesity. Adipocyte differentiation, a critical event in the progression of obesity, is an intricate process tightly controlled by various transcription factors, including the members of the CCAAT/enhancer binding protein (C/EBP) family and peroxisome proliferator-activated receptor γ (PPARγ) [10,11]. C/EBPβ and C/EBPδ are important regulators of the initial phases of adipocyte differentiation; C/EBPβ allows the growth-arrested preadipocytes to reinitiate mitotic clonal expansion (MCE) [12,13]. Then, C/EBPβ and C/EBPδ synergistically promote the expression of C/EBPα and PPARγ upon stimulation with a differentiation cocktail [14]. PPARγ is considered to be the dominant inducer of adipocyte differentiation due to its indispensable role in adipogenesis; its absence is sufficient to inhibit adipocyte differentiation [15,16]. C/EBPα, a critical downstream effector of PPARγ, maintains the expression of PPARγ and functionally synergizes with PPARγ to induce the expression of adipogenic genes functioning in the late stages of adipocyte differentiation, such as adiponectin and fatty acid binding protein 4 (FABP4, also known as aP2) [17,18].

Metallothioneins (MTs) are metal-binding proteins of low molecular weight (6–7 kDa) with a high cysteine content (30%). The four major MTs are MT1, MT2, MT3, and MT4 [19,20,21]. Among them, MT1 and MT2 are widely expressed in mammals, whereas MT3 is primarily found in the brain, and MT4 is expressed only in certain squamous epithelia [19,22]. MTs are associated with multiple physiological functions, including detoxification of heavy metals, maintenance of metal ion homeostasis (especially zinc and copper), and protection against DNA damage and oxidation [23,24,25,26]. MTs play key roles in signaling pathways relevant to several disease conditions, including cancer and neurodegenerative diseases [27,28]. Multiple studies have shown that MTs are also closely associated with obesity. *MT1/2*-knockout male mice fed a high-fat diet (HFD) displayed enhanced features of obesity (increased fat accumulation and obese (*ob*) gene expression) compared with wide mice [29]; this finding was further confirmed in the *MT1/2*-knockout female mice [30]. *MT3*-knockout male mice also exhibited elevated weight gain with aging compared with wild-type mice, which was associated with reduced levels of leptin receptors [31].

Reactive oxygen species (ROS) are essential mediators that are not only linked to aging and pathological conditions, such as cancer, diabetes, and obesity, but are also required for multiple physiological processes essential for life [32,33]. Previous studies have indicated that oxygen consumption and production of intracellular and mitochondrial ROS increase during 3T3-L1 adipocyte differentiation [34,35]. In addition, oxidative stress was found to induce the accumulation of lipid droplets through SREBP1c activation in HepG2 cells [36]. Moreover, ROS can promote adipocyte differentiation in 3T3-L1 preadipocytes, which is mediated by advancing MCE [37]. These observations indicate that ROS act as crucial factors in adipocyte differentiation. Intriguingly, the ROS scavenging activity of MT3, which is rich in cysteine residues with a high potential to interact with ROS, is considered to be one of its major functions [38], suggesting that MT3 may be closely related to adipocyte differentiation.

In this study, we first found that MT3 was downregulated in the process of 3T3-L1 adipocyte differentiation. To investigate the role of MT3 in this process, we overexpressed MT3 in 3T3-L1 cells. Our data revealed that MT3 can suppress 3T3-L1 adipocyte differentiation indirectly by inhibiting the transcriptional activity of PPARγ and by reducing ROS levels in the early stages of adipogenesis, thus providing a potential novel target for the prevention and treatment of obesity.

## 2. Materials and Methods

### 2.1. Cell Culture

Mouse preadipocyte 3T3-L1 cells and human embryonic kidney (HEK) 293T cells were obtained from the American Type Culture Collection. The 3T3-L1 cells were cultured in Dulbecco’s modified Eagle medium (DMEM, #12100046, Gibco™, Carlsbad, CA, USA) supplemented with 10% bovine calf serum (BCS, Welgene Inc., Daegu, Republic of Korea) and 1% antibiotic–antimycotic (#15240062; Gibco™). HEK 293T cells were maintained in DMEM containing 10% fetal bovine serum (FBS, Welgene Inc.) and 1% antibiotic–antimycotic at 37 °C in a humidified incubator with 5% CO_2_. 

### 2.2. 3T3-L1 Adipocyte Differentiation

After reaching confluence, the 3T3-L1 cells were maintained for 48 h. Then, the growth medium was replaced with the differentiation medium, consisting of DMEM supplemented with 10% FBS and a differentiation cocktail with 0.5 mM 3-isobutyl-1-methylxanthine (Sigma, St. Louis, MO, USA), 10 μg/mL insulin (Sigma), and 1 μM dexamethasone (Sigma) on day 0. Two days after hormonal induction (on day 2), the differentiation medium was replaced with DMEM supplemented with 10% FBS and 10 μg/mL insulin. The media was replaced every two days with DMEM supplemented with 10% FBS. On day 8, the lipid droplets were observed in the cells, which were used for further experiments.

### 2.3. Plasmids and Transfection

The HA-tagged MT3 plasmid or Myc-tagged PPARγ plasmid were constructed in a CMV promoter-derived mammalian expression vector (pCS4+). For MT3 knockdown experiments, oligonucleotides for small hairpin RNA (shRNA) were generated by targeting a 19-base pair sequence of the mouse MT3 gene. shMT3#1: sense 5′-GAT CCC CCC AAG GAC TGT GTG TGC AAT TCA AGA GAT TGC ACA CACA GTC CTT GGT TTTTA-3′, antisense 5′-AGC TTA AAA ACC AAG GAC TGT GTG TGC AAT CTC TTG AAT TGC ACA CAC AGT CCT TGG GGG-3′; shMT3#2: sense 5′-GAT CCC CGC AAG TGC AAG GGC TGC AAA TTT CAA GAG AAT TTG CAG CCC TTG CAC TTG C TTTTTA-3′, antisense 5′-AGC TTA AAA AGC AAG TGC AAG GGC TGC AAA TTC TCT TGA AAT TTG CAG CCC TTG CAC TTG CGGG-3′. Sense and antisense oligonucleotides were annealed and ligated into a pSuper-retro vector (Oligoengine, Seattle, WA, USA). Both overexpression and knockdown experiments in the HEK 293T cells and 3T3-L1 cells were performed by using polyethyleneimine (PEI) (Polysciences, Inc., Warrington, PA, USA).

### 2.4. Oil Red O Staining

Oil Red O staining was performed to measure the extent of adipocyte differentiation. Briefly, cells were washed two times with phosphate-buffered saline (PBS) and fixed with 10% formalin at room temperature for 30 min. Next, the cells were washed with PBS twice and incubated with 60% isopropanol for 1 min. Then, 250 μL (for a 24-well plate) of 0.5% Oil Red O staining solution (O0625; Sigma, St. Louis, MO, USA) was added to each well to cover the cell monolayer, and the cells were incubated at room temperature for 25 min on a shaker. The staining solution was carefully aspirated, and the cells were washed with PBS three times. An inverted microscope and NIS-Elements software (Niko Company, Fukuoka, Japan) were used to visualize lipid droplet accumulation. For the quantification of lipid accumulation, the stain was extracted in isopropanol, and the absorbance was measured at 510 nm using an Epoch microplate reader (BioTek Company, New Castle, DE, USA).

### 2.5. Triglyceride (TG) Colorimetric Assay

For cellular triglyceride determination, fully differentiated 3T3-L1 cells were washed twice with PBS and collected by cell scraper. They were centrifuged at 1000× *g* for 10 min, and then the supernatant was removed and the cell sediment was retained. The cells added isopropanol into the sediment according to the cell number (2 × 10^6^) ratio: isopropanol (μL) = 1:200. Then, centrifuging was performed at 10,000× *g* for 10 min at 4 °C, and the supernatant was taken for detention. Cellular triglyceride content was determined by the TG Colorimetric Assay Kit (E-BC-K261-M; Elabscience Biotechnology Inc., Houston, TX, USA).

### 2.6. Luciferase Reporter Assays

HEK 293T cells were seeded in a 24-well plate and transfected with combinations of plasmids expressing indicated proteins (MT3 and PPARγ), luciferase reporters (aP2-Luc or PPRE-Luc), and CMV promoter-driven β-galactosidase (β-gal). PPRE-Luc includes the consensus PPAR response element (PPRE), and aP2-Luc contains a promoter region of adipocyte aP2 that includes PPREs [39]. A β-gal plasmid was used to monitor transfection efficiency. The cells were treated with the PPARγ agonist rosiglitazone 24 h after transfection and were lysed to determine the activities of luciferase reporters using a luciferase reporter assay kit (Promega, Madison, WI, USA).

### 2.7. Immunoblotting

Cells were lysed in an ice-cold lysis buffer (1% NP-40, 25 mM HEPES at pH 7.5, 10% glycerol, 0.25% sodium deoxycholate, 1 mM EDTA, 1 mM Na_3_VO_4_, 25 mM NaF, 150 mM NaCl, 10 mg/mL aprotinin, 10 mg/mL leupeptin, and 250 mM phenylmethanesulfonyl fluoride). For immunoblotting (IB), the samples (50–80 μg of total protein per sample) were run on sodium dodecyl sulfate–polyacrylamide gel electrophoresis (SDS-PAGE) and then transferred onto polyvinylidene fluoride membranes. Then, membranes were incubated with primary antibodies at 4 °C overnight. The primary antibodies used are shown in Table 1. After incubation with appropriate horseradish peroxidase-conjugated secondary antibodies, an Immobilon Western Chemiluminescent HRP Substrate (WBKLS0500, Millipore, Billerica, MA, USA) was used to visualize the protein bands, and the images were captured using an Amersham^TM^ ImageQuant^TM^ 800 system (GE Healthcare Life Sciences, Marlborough, MA, USA).

### 2.8. Immunoprecipitation

For immunoprecipitation, supernatants of centrifuged lysates were incubated with appropriate antibodies at 4 °C overnight on a shaker. Next, protein A-Sepharose beads (17096303, GE Healthcare) were added (40 μL per sample). The samples were incubated for 2 h at 4 °C, centrifuged (3000 rpm) to precipitate the beads, and washed with lysis buffer three times at 4 °C. Finally, the supernatant was removed, and 20 µL of 5× loading buffer (20 mL of glycerol, 25 mL of 10% SDS, 5 mL of 1 M tris at pH 7.5, 100 µL of Bromo blue phenol, and 50 mL of double distilled water) was added to each sample. Then, the samples were boiled for 5 min at 100 °C. The immunoprecipitated proteins were subsequently subjected to SDS-PAGE and visualized by immunoblotting.

### 2.9. Reverse Transcription followed by Quantitative PCR (RT-qPCR)

The total RNA from the 3T3-L1 cells was extracted using an RNAiso Plus kit (TaKaRa, Tokyo, Japan), and cDNA was synthesized using Oligo (dT) primers with GoScript^TM^ Reverse Transcription System (Promega). Quantitative PCR was performed using a TB Green^®^ Premix Ex Taq™ (Tli RNaseH Plus) Kit (TaKaRa) following the manufacturer’s protocol. The primer sequences for the target genes are summarized in Table 2.

### 2.10. Dihydroethidium (DHE) Staining

The DHE Assay Kit—Reactive Oxygen Species (Abcam, Cambridge, UK) was used to determine intracellular ROS levels in the 3T3-L1 cells. The cells seeded in 96-well plates were transfected with plasmids inducing MT3 overexpression or knockdown. Upon confluence, the cells were incubated with antimycin A or N-acetyl cysteine (NAC) for 12 h. Next, the 3T3-L1 cells were incubated with DHE buffer at 37 °C for 1 h, and images were captured using a fluorescence microscope and a digital camera.

### 2.11. Statistical Analysis

GraphPad Prism 8.0.2 was used for statistical analysis. One-way ANOVA followed by Tukey’s Test (to compare the mean of each group with the mean of every other group) or Dunnett’s Test (to compare the mean of each group with the control group) was used to evaluate the differences. All experiments were repeated at least three times. The data are expressed as the means ± SEM, and differences with *p*-values smaller than 0.05 were considered significant.

## 3. Results

### 3.1. MT3 Is Significantly Downregulated during 3T3-L1 Adipocyte Differentiation

**Figure 1:**
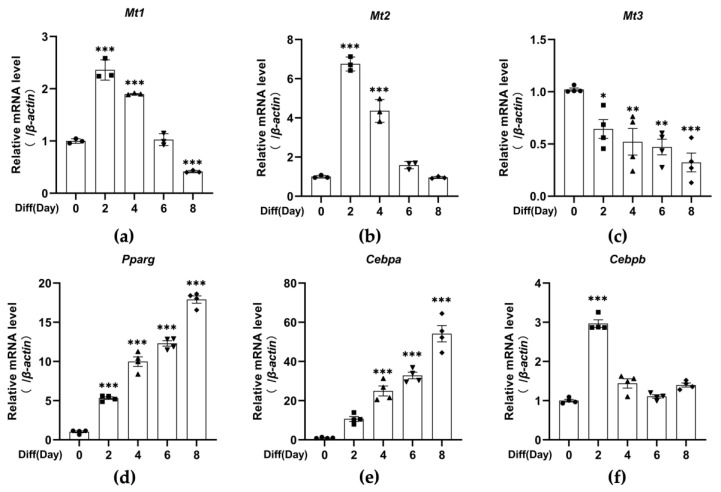
The mRNA levels of *Mt3* are significantly decreased during 3T3-L1 adipocyte differentiation. The differentiation of 3T3-L1 adipocytes was induced by the differentiation cocktail. (**a**–**f**) RT-qPCR analysis was used to detect the mRNA levels of *Mt1*, *Mt2*, *Mt3*, *Pparg*, *Cebpa*, and *Cebpb* at specific times (days 0, 2, 4, 6, and 8). *β-actin* served as a loading control. The data are presented as the means ± SEM. * *p* < 0.05, ** *p* < 0.01, *** *p* < 0.001 vs. the corresponding control group (day 0) were calculated by one-way ANOVA followed by Dunnett’s Test.

**Figure 2:**
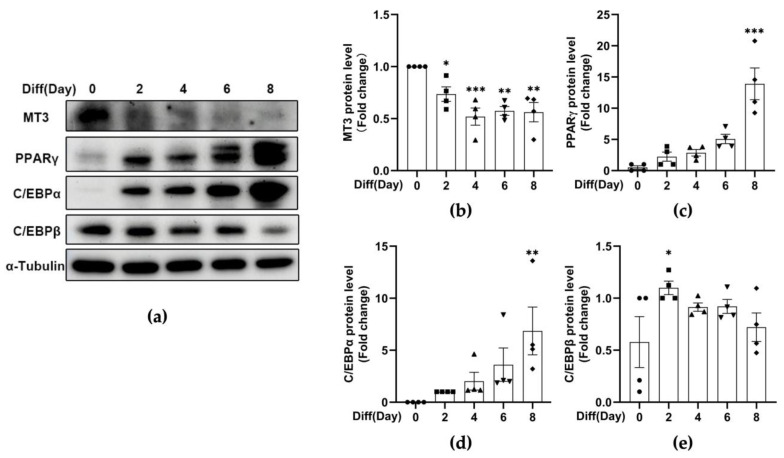
The protein levels of MT3 are significantly decreased during 3T3-L1 adipocyte differentiation. The differentiation of 3T3-L1 adipocytes was induced by the differentiation cocktail. (**a**) Immunoblotting was used to detect the protein levels of MT3, C/EBPα, C/EBPβ, PPARγ, and α-Tubulin at specified times (days 0, 2, 4, 6, and 8). α-Tubulin was used as a loading control. (**b**–**e**) The intensities of bands (MT3, C/EBPα, C/EBPβ, and PPARγ) were quantified and normalized with the corresponding α-Tubulin bands. The data are presented as the means ± SEM. * *p* < 0.05, ** *p* < 0.01, *** *p* < 0.001 vs. the corresponding control group (day 0) were calculated by one-way ANOVA followed by Dunnett’s Test.

Previous studies in our lab have demonstrated that MT3 is important for osteoblast differentiation [40], and numerous in vitro studies have shown that osteogenic factors inhibit adipogenesis, while adipogenic factors hinder osteogenesis [41,42,43]. Therefore, to explore the role of MT3 in adipocyte differentiation, we induced 3T3-L1 adipocyte differentiation and examined the levels of *Mt1*, *Mt2*, and *Mt3* by RT-qPCR. The induction of adipocyte differentiation was successful, as indicated by the expression of the early-stage marker C/EBPβ and the late-stage adipogenesis markers C/EBPα and PPARγ (Figure 1d–f and Figure 2a,c–e). We found that the mRNA levels of *Mt1* and *Mt2* peaked on day 2 and then gradually decreased afterward, while the mRNA levels of *Mt3* dramatically decreased during the period of adipocyte differentiation (Figure 1a–c). The protein levels of MT3 were reduced in a similar manner (Figure 2a,b). These findings suggest that MT3 might play a role in 3T3-L1 adipocyte differentiation.

### 3.2. Mt3 Overexpression Inhibits Lipid Accumulation in 3T3-L1 Adipocytes

**Figure 3:**
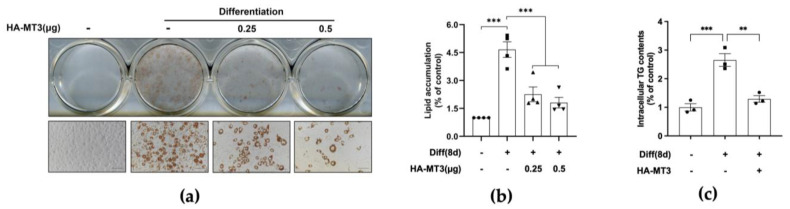
*Mt3* overexpression inhibits lipid accumulation in 3T3−L1 adipocytes. 3T3−L1 cells were transfected with HA−MT3 plasmid (0.25, 0.5 μg) prior to contact inhibition and incubated with differentiation cocktail to induce adipocyte differentiation. Oil Red O staining was used to measure the extent of lipid accumulation on day 8. (**a**) Representative images of 3T3-L1 adipocytes were captured at 200× magnification. Scale bar = 100 μm. (**b**) The lipid accumulation in 3T3−L1 adipocytes was quantified as a percentage of control values. (**c**) Intracellular triglyceride content was measured by using a commercial kit. The data are presented as the means ± SEM. ** *p* < 0.01, *** *p* < 0.001 vs. the corresponding undifferentiated and differentiated groups were calculated by one-way ANOVA followed by Tukey’s Test.

To further investigate the potential function of MT3 in 3T3-L1 adipocyte differentiation, we overexpressed *Mt3* in 3T3-L1 preadipocytes before they reached confluence. The formation and accumulation of lipid droplets are considered the predominant characteristic of mature adipocyte differentiation; thus, Oil Red O staining was used to assess the extent of adipocyte differentiation. Microscopy images showed that the differentiated control group acquired the phenotype of mature adipocyte after 8 days of differentiation, and *Mt3* overexpression significantly decreased the intracellular lipid accumulation, which was confirmed by quantitative analysis (Figure 3a,b). Furthermore, Mt3 overexpression also evidently decreased intracellular triglyceride content (Figure 3c). Taken together, these results demonstrate that MT3 can inhibit 3T3-L1 adipocyte differentiation.

### 3.3. Mt3 Overexpression Suppresses the Protein Levels of Adipogenic Transcriptional Factors in 3T3-L1 Cells

**Figure 4:**
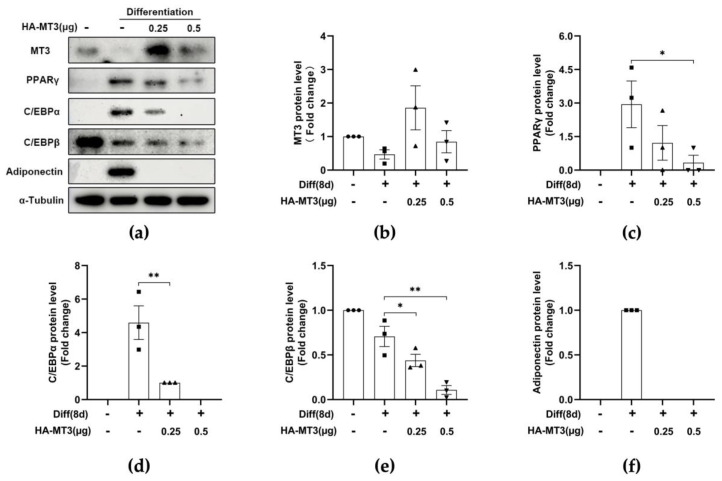
*Mt3* overexpression reduces the protein levels of adipogenic transcription factors in 3T3-L1 adipocytes. 3T3−L1 cells were transfected with HA−MT3 plasmid (0.25, 0.5 μg) prior to contact inhibition and induced adipocyte differentiation using differentiation cocktail. (**a**) The protein levels of MT3, PPARγ, C/EBPα, C/EBPβ, adiponectin, and α-Tubulin in the different groups were measured by immunoblotting. α−Tubulin was used as a loading control. (**b**–**f**) The intensities of bands (MT3, C/EBPα, C/EBPβ, PPARγ, and adiponectin) were quantified and normalized with the corresponding α-Tubulin bands. The data are presented as the means ± SEM. * *p* < 0.05, ** *p* < 0.01 were calculated by one-way ANOVA followed by Tukey’s Test.

The sequential expression of genes associated with the specific characteristics of adipocytes takes place during adipocyte differentiation. Therefore, we investigated whether the decrease in the accumulation of lipid droplets in 3T3-L1 cells was due to a downregulation of adipogenic transcription factors. Immunoblotting analysis revealed that the protein levels of the early-stage marker C/EBPβ and the late-stage markers C/EBPα and PPARγ were all reduced in cells overexpressing *Mt3* (Figure 4a–e). Adiponectin is an adipokine that regulates a variety of metabolic events, including fatty acid oxidation and glucose levels, and exhibits the highest mRNA expression levels in adipocytes [44]. In our study, the protein levels of adiponectin were also dramatically decreased by *Mt3* overexpression compared with the differentiated group (Figure 4f). These data demonstrate that MT3 inhibits adipocyte differentiation by attenuating the expression of adipogenic transcription factors.

### 3.4. Mt3 Overexpression Reduces Adipogenesis-Related Gene Expression in 3T3-L1 Cells

**Figure 5:**
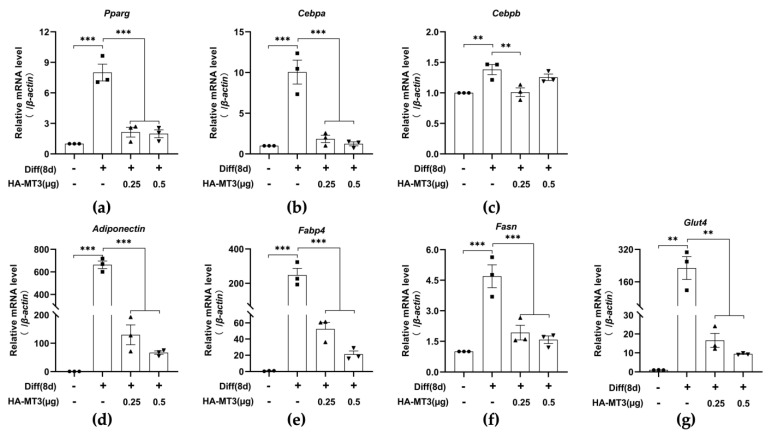
*Mt3* overexpression reduces adipogenesis-related gene expression in 3T3−L1 cells. 3T3−L1 cells were transfected with HA−MT3 plasmid (0.25, 0.5 μg) prior to contact inhibition and induced adipocyte differentiation using differentiation cocktail. (**a**–**g**) The mRNA levels of *Mt3*, *Pparg*, *Cebpa*, *Cebpb*, *Adiponectin*, *Fabp4*, *Fasn,* and *Glut4* in the different groups were compared by RT-qPCR. *β-actin* was used as a loading control. The data are presented as the means ± SEM. ** *p* < 0.01, *** *p* < 0.001 were calculated by one-way ANOVA followed by Tukey’s Test.

As *Mt3* overexpression resulted in a downregulation of the protein levels of adipogenic transcription factors, we further examined the mRNA levels of genes encoding these factors. Similar to the protein levels, *Mt3* overexpression significantly inhibited the mRNA levels of adipogenesis-related genes, such as *Pparg*, *Cebpa*, *Cebpb*, and *Adiponectin* (Figure 5a–d). In addition, we assessed the expression levels of other adipogenesis-related genes associated with lipogenesis, fatty acid oxidation, and glucose homeostasis pathways [45,46]. Upon differentiation induction, the mRNA levels of fatty acid synthase (*Fasn*), *Fabp4*, and glucose transporter type 4 (*Glut4*) were highly elevated compared with the undifferentiated group, whereas *Mt3* overexpression dramatically reduced these increases (Figure 5e–g).

### 3.5. MT3 Indirectly Suppresses PPARγ Transcriptional Activity

**Figure 6:**
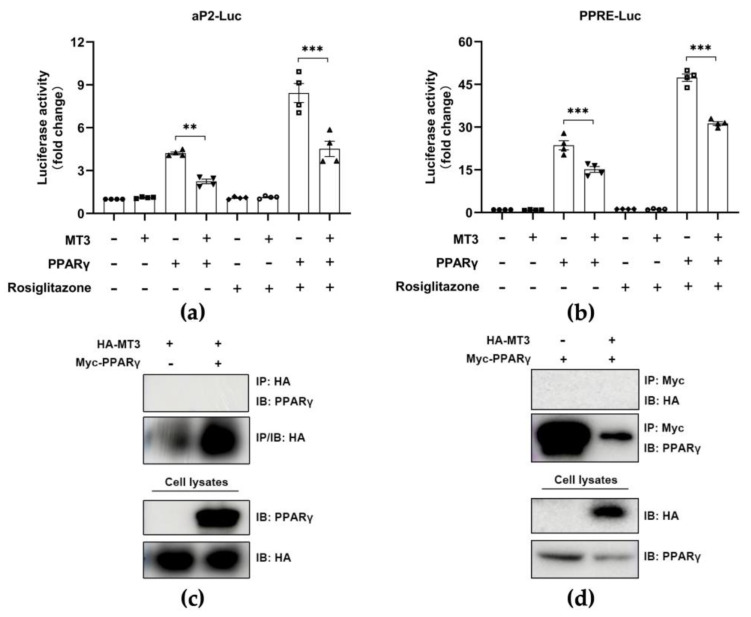
MT3 indirectly suppresses PPARγ transcriptional activities. (**a**,**b**) HEK 293T cells were transfected with indicated HA−MT3 (0.5 μg) or combinations of HA−PPARγ (0.125 μg) and HA-MT3 (0.5 μg) along with aP2−Luc or PPRE-Luc reporter. pCMV−β−gal (0.025 μg) was used to normalize the transfection efficiency. HEK 293T cells were incubated with 0.5 μM rosiglitazone and DMSO 24 h after transfection and cultured for an additional 24 h. The luciferase reporter activities were measured following 48 h transfection. The data are presented as the means ± SEM. ** *p* < 0.01, *** *p* < 0.001 were calculated by one-way ANOVA followed by Tukey’s Test. (**c**,**d**) HEK 293T cells were transfected with indicated expression plasmids (HA−MT3, Myc−PPARγ, or empty Myc vector) for immunoprecipitation.

PPARγ is considered the most important regulator of adipocyte differentiation [15,16]. Thus, we investigated whether MT3 regulated the transcriptional activity of PPARγ by performing a luciferase reporter assay to measure the transcriptional activity of PPARγ in HEK 293T cells. As expected, PPARγ alone stimulated the activity of luciferase reporters, and MT3 did not affect the activity of luciferase reporters. However, we observed that *Mt3* overexpression evidently inhibited the activity of luciferase reporters in the presence of *Pparg* overexpression, while this inhibitory effect was more pronounced following the incubation with rosiglitazone, an agonist of PPARγ (Figure 6a,b). These results suggested that *Mt3* overexpression suppressed PPARγ transcriptional activity. Then, we examined whether there was an interaction between MT3 and PPARγ in HEK 293T cells by performing immunoprecipitation. However, we did not detect an interaction between MT3 and PPARγ (Figure 6c,d). Taken together, these data suggested that MT3 downregulated PPARγ transcriptional activity not by interacting with PPARγ but through an indirect mechanism.

### 3.6. MT3 Impedes ROS Production in the Early Stage of 3T3-L1 Adipocyte Differentiation

**Figure 7:**
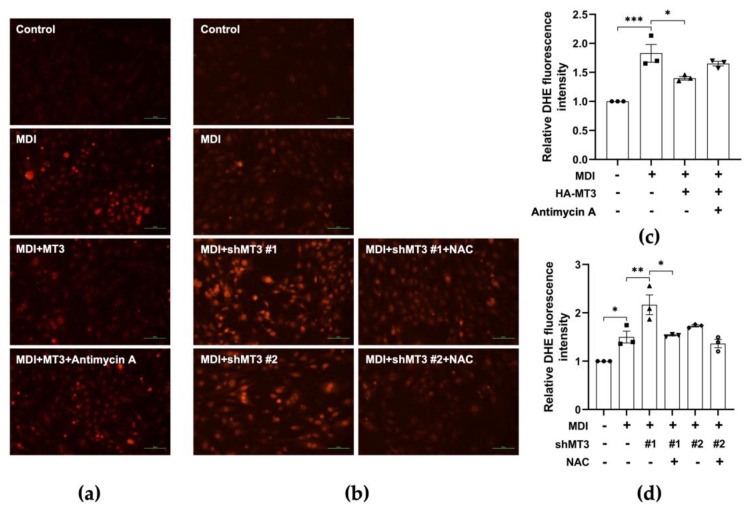
MT3 impedes ROS production in the early stages of 3T3−L1 adipocyte differentiation. (**a**,**b**) 3T3−L1 cells were transfected with HA−MT3 plasmid or shMT3 plasmid (0.2 μg) prior to contact inhibition and cultured in differentiation medium with 25 nM antimycin A or 7.5 mM NAC for 12 h. ROS levels are measured by DHE assay. Representative images of 3T3-L1 adipocytes were captured at 200× magnification. Scale bar = 100 μm. MDI, a mixture of 3-isobutyl-1-methylxanthine, dexamethasone, and insulin in differentiation medium. (**c**,**d**) The quantitative analysis of the relative DHE fluorescence intensity indicated the differences in each group. The data are presented as the means ± SEM. * *p* < 0.05, ** *p* < 0.01, and *** *p* < 0.001 were calculated by one-way ANOVA followed by Tukey’s Test.

Given that *Mt3* overexpression downregulated the levels of adipogenic transcription factors including PPARγ but indirectly regulated PPARγ transcriptional activity, we sought to identify the mechanism underlying the inhibitory effect of MT3 on 3T3-L1 adipocyte differentiation. MT3 is a powerful scavenger of ROS due to its particular structure [38]. In addition, the early stages of adipocyte differentiation are associated with elevated ROS levels [37], implying that ROS plays a role in adipocyte differentiation. Therefore, we hypothesized that MT3 might affect ROS generation during adipocyte differentiation. To test our hypothesis, we performed DHE staining to measure ROS levels in 3T3-L1 cells. We found that *Mt3* overexpression decreased the elevated ROS levels induced by differentiation, while antimycin A treatment reversed the MT3-induced decreases in ROS levels (Figure 7a,c). Antimycin A is a mitochondrial respiratory chain inhibitor that is experimentally used to induce ROS generation [47]. To further examine the effect of MT3 on ROS levels, we knocked down MT3 expression by transfecting the cells with shRNA targeting *Mt3*. Compared with the differentiated control group, *Mt3* knockdown dramatically elevated ROS levels. However, treatment with NAC, an ROS scavenger, attenuated the *Mt3* knockdown-induced increases in ROS levels (Figure 7b,d). Taken together, these results indicate that MT3 can impede ROS production in the early stages of adipocyte differentiation.

## 4. Discussion

MTs are multipurpose proteins with essential roles in a variety of pathological conditions. Previous studies have demonstrated that MTs are potentially involved in obesity and its complications. MTs are secreted by white adipose tissue in mice [48], but their functions in the adipose tissue are not clear. Clinical studies have shown that MT1A and MT2A levels are upregulated in subcutaneous and visceral adipose tissues of patients with obesity or type 2 diabetes [49,50], implying that increased expression of MTs in human adipose tissues may be either a factor contributing to the development of obesity or a consequence of obesity. A recent study further reported the regulatory effect of MT1/2 on sex-specific differences observed in HFD-induced obesity. MTs can enhance the activity of androgens to promote fat storage and the function of estrogen in preventing excess fat accumulation [51]. In fact, *Mt3*-knockout mice fed an HFD were more susceptible to weight gain compared with *Mt1/2*-knockout mice fed an HFD [52]. However, there are no clear data on how MT3 is engaged in the development of obesity, especially in 3T3-L1 adipocyte differentiation; the underlying molecular pathways remain unclear.

A previous study showed that MTs are not secreted during the maintenance of fibroblast-like preadipocytes but are released after the induction of differentiation by exposure to a “differentiation cocktail”. Furthermore, the release of MTs into the culture medium precedes leptin expression [48], indicating that MTs may be potential markers of adipocyte differentiation. Yoshito Kadota et al. [53] also describe the expression patterns of MT1 and MT2 during 3T3-L1 adipocyte differentiation. In our study, we further examined the expression patterns of MT3 during 3T3-L1 adipocyte differentiation. We observed that MT3 expression was significantly decreased during differentiation, along with the upregulation of PPARγ and C/EBPα. The Oil Red O staining results demonstrated that MT3 repressed the accumulation of lipid droplets, suggesting that MT3 may inhibit adipocyte differentiation. This was further confirmed by the downregulation of the adipogenic transcription factors PPARγ and C/EBPα. Considering that adipocyte differentiation is a multi-step process, we examined the protein levels of related markers, including PPARγ, C/EBPα, and C/EBPβ, at different stages of differentiation to clarify the steps in which MT3 may significantly affect differentiation. We found that MT3 reduced the protein level of PPARγ and C/EBPα at specific times (day 2, day 4, day 6, and day 8) (Figure S1). The differentiation of mature adipocytes from preadipocytes is involved in the successive activation of a range of transcription factors, among which PPARγ is the most important one [54]. Considering the central role of PPARγ in adipocyte differentiation, we hypothesized that MT3 might mediate the PPARγ transcriptional activity. To this end, a luciferase reporter assay was performed to measure its transcriptional activity. As suspected, MT3 suppressed PPARγ transcriptional activity, and the inhibitory effect of PPARγ was more pronounced after treatment with the PPARγ agonist rosiglitazone. We considered the possibility that the inhibitory effect of MT3 on PPARγ transcriptional activity was due to a potential interaction between MT3 and PPARγ. However, immunoprecipitation assays indicated that MT3 did not bind PPARγ. Therefore, the mechanism through which MT3 regulates PPARγ transcriptional activity is still unclear; one possibility is that MT3 may interact with upstream factors of PPARγ, such as C/EBPβ, or another important factor, C/EBPα, which needs to be further investigated.

As in the case of other MT family members, ROS scavenging ability is the most important function of MT3 [55]. Both intracellular and extracellular MT3 protect neurons from oxidative damage under stress-induced conditions due to their function in the elimination of ROS [56,57], which plays a potential role in adipocyte differentiation. Elevated ROS levels during the initial stages of adipocyte differentiation are observed in both 3T3-L1 cells and mesenchymal stem cells (10T1/2), indicating that ROS generation is essential for adipocyte differentiation. In line with this idea, adipocyte differentiation is promoted by H_2_O_2_ treatment but inhibited by the antioxidant NAC [37,58]. ROS can originate from mitochondrial complex III and can regulate the differentiation process from the primary human mesenchymal stem cells into adipocytes, which is mainly dependent on mTORC1 signaling [59]. In adipose-derived stem cells, hypoxia (2% oxygen) enhances the differentiation through the generation of mitochondrial ROS [60]. Hence, we asked whether there was a relationship between MT3, ROS levels, and adipocyte differentiation. Here, we found that ROS were induced in the initial phase of 3T3-L1 adipocyte differentiation. We also observed that *Mt3* overexpression could impede ROS generation during differentiation; on the contrary, *Mt3* knockdown significantly elevated ROS production; importantly, these effects were largely reversed by treatments with the ROS inducer antimycin A and the antioxidant NAC, respectively. In addition, *Mt3* overexpression could reduce the MDI-induced increase of ROS scavenging genes, such as *Catalase*, *Gpx1*, *Sod1*, and *Sod2*, while antimycin A treatment reversed this effect (Figure S2). To further elucidate whether MT3 could regulate adipocyte differentiation through its powerful antioxidant effect, we treated *Mt3*-overexpressing 3T3-L1 cells with or without antimycin A. The Oil Red O staining results showed that antimycin A could partially restore the inhibitory role of MT3 on 3T3-L1 adipocyte differentiation (Figure S3). Taken together, these results suggest that MT3 hinders 3T3-L1 adipocyte differentiation largely by attenuating the ROS levels during the early stages of adipocyte differentiation.

A previous study reported a partial relationship between ROS levels and C/EBPβ activity with the following observations: (i) the initial phase of adipocyte differentiation with elevated ROS levels corresponds to the S phase of MCE, when C/EBPβ is translocated into centromeres; (ii) antioxidant treatment not only blocks ROS production but also prevents the translocation of C/EBPβ. Therefore, this study [37] demonstrated that ROS is essential for the MCE of 3T3-L1 preadipocytes, which is closely linked to the DNA binding activity of C/EBPβ. Furthermore, there are some other studies showing that ROS levels can be reduced by PPARγ and its agonists [61,62,63]. However, we did not investigate the connection between the decreased ROS levels caused by MT3 and the adipogenic transcription factors C/EBPβ and PPARγ, which should be further studied in the future.

Adipocyte differentiation is a complex process regulated by various signaling pathways. For example, the mitogen-activated protein kinase (MAPK) signaling pathway plays a pivotal role in adipocyte differentiation [64]. Extracellular signal-regulated kinases (ERKs), c-Jun amino-terminal kinases (JNKs), and p38 MAPK are the three main important subfamilies of the MAPK signaling pathway [65]. ERKs can be activated by mitogens including growth factors or serum, and its activation is essential for MCE, which is indispensable for early adipocyte differentiation [66]. Conversely, phosphorylated Erk1/2 inhibits 3T3-L1 adipocyte differentiation through a reduction in PPARγ transcriptional activity [67]. These opposite effects of ERKs might be associated with the different stages of differentiation. JNK2 has a positive impact on 3T3-L1 adipocyte differentiation, and its activation specifically contributes to the initial stage of differentiation, as JNK inhibition cannot affect terminal differentiation [68]. p38 MAPK inhibitors function to block 3T3-L1 adipocyte differentiation during the early stage of differentiation [69]. This study also pointed out that C/EBPβ serves as a substrate for p38 MAPK in vitro due to its consensus site for p38 phosphorylation. In addition, the MAPK signaling pathway is closely linked to the ROS pathway. ERK1/2 has been reported as the potential downstream target of ROS in inflammation-related diseases [70,71]. ROS can induce the activation of the MAPK signaling pathway, and the decreased accumulation of ROS by antioxidants suppresses MAPK activation, implying that ROS play a vital role in the activation of MAPK pathways [72,73]. The phosphatidylinositol 3-kinase (PI3K)/Akt signaling pathway also plays a critical role in adipocyte differentiation [74]. The constitutively active form of Akt promotes 3T3-L1 adipocyte differentiation [75], and ROS are known to enhance Akt phosphorylation [76]. A previous study showed that mTORC1 signaling is required for the early increase in ROS during adipocyte differentiation of human mesenchymal stem cells [59]. In addition, ROS can activate AMP-activated protein kinase (AMPK) and further induce the DNA binding activity of C/EBPβ, leading to the promotion of adipogenesis [77].

Furthermore, the generation of complex III ROS in the mitochondria, which induces the PPARγ transcriptional machinery, is required for adipocyte differentiation [59]. On the basis of our finding that MT3 can suppress the expressions of PPARγ and its target genes, we assume that the inhibition of ROS generation by MT3 might suppress the PPARγ transcriptional machinery and consequently inhibit 3T3-L1 adipocyte differentiation. However, the detailed mechanism of how the decrease in ROS via MT3 regulates PPARγ transcriptional machinery remains elusive.

## 5. Conclusions

**Figure 8:**
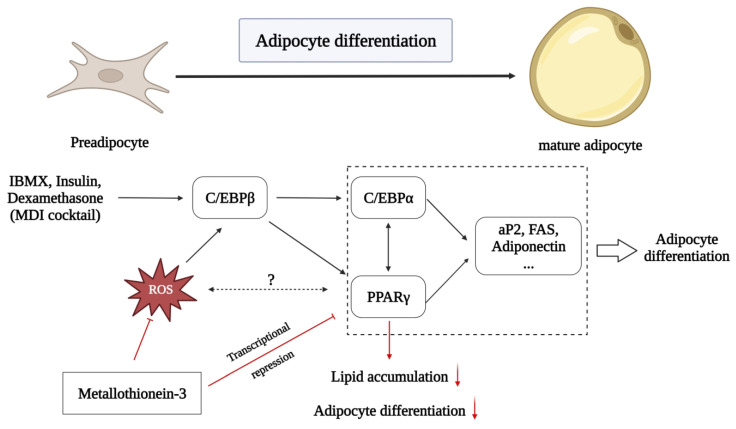
Schematic diagram showing the regulatory effect of MT3 in 3T3-L1 adipocyte differentiation. Adipocyte differentiation is a complex physiological process involved in various transcription factors, including the C/EBP family and PPARγ. On one hand, MT3 can inhibit 3T3-L1 adipocyte differentiation by indirectly suppressing the PPARγ transcriptional activity. On the other hand, the inhibitory effect of MT3 on 3T3-L1 adipocyte differentiation is largely dependent on reducing ROS levels in the initial adipocyte differentiation. The red arrows represent new findings from our study, while the blank arrows stand for previously known effects.

In conclusion, our findings suggest a new role for MT3 in the differentiation of 3T3-L1 cells into adipocytes (Figure 8). Our results indicate that MT3 acts as a novel inhibitor of adipocyte differentiation. MT3 can suppress the levels of adipogenic transcription factors such as C/EBP family members and PPARγ. MT3 also downregulates the transcriptional activity of PPARγ. Furthermore, the ability of MT3 to regulate adipocyte differentiation is largely dependent on its ROS scavenging activity. Although our findings are limited in vitro, this study might provide a potential target for the prevention and treatment of obesity.

## Figures and Tables

**Table 1:** The primary antibodies used for immunoblotting.

Antibody	Order#	Company	Dilution
MT3	LS-C295361	LifeSpan BioSciences, Seattle, WA, USA	1:400
PPARγ	D69	Cell Signaling Technology, Danvers, MA, USA	1:1000
C/EBPα	SC-61	Santa Cruz Biotechnology, Santa Cruz, CA, USA	1:1000
C/EBPβ	SC-150	Santa Cruz Biotechnology, Santa Cruz, CA, USA	1:1000
adiponectin	C45B10	Cell Signaling Technology, Danvers, MA, USA	1:1000
α-Tubulin	sc-53646	Santa Cruz Biotechnology, Santa Cruz, CA, USA	1:1000
HA	12CA5	Roche Applied Science, Basel, Switzerland	1:1000
Myc	9E10	Santa Cruz Biotechnology, Santa Cruz, CA, USA	1:1000

**Table 2:** Primer sequences used for RT-qPCR.

Gene	Accession Number	Forward Primer (5′–>3′)	Reverse Primer (5′–>3′)
*Mt1*	NM_013602.3	AAGAGTGAGTTGGGACACCTT	CGAGACAATACAATG GCCTCC
*Mt2*	NM_008630.2	ATGCAAATGTACTTCCTGCAAGA	CTGGGAGCACTTCGCACAG
*Mt3*	NM_013603.2	TGCACCTGCTCGGACAAAT	CTTGGCACACTTCTCACATC
*Pparg*	NM_011146.4	GCCCTTTGGTGACTTTATGGA	GCAGCAGGTTGCTTGGATG
*Cebpa*	NM_007678.3	CAAGAACAGCAACGAGTACCG	GTCACTGGTCAACTCCAGCAC
*Cebpb*	NM_001287738.1	ACCGGGTTTCGGGACTTGA	GTTGCGTCAGTCCCGTGTCCA
*Fasn*	NM_007988.3	GCTATGCAGATGGCTGTCTCTCCCAG	GCAGCGCTGTTTACATTCCTCCCAGG
*Glut4*	NM_001359114.1	GTAACTTCATTGTCGGCATGG	AGCTGAGATCTGGTCAAACG
*Adiponectin*	NM_009605.5	CATCCCAGGACATCCTGGCCACAATG	GGCCCTTCAGCTCCTGTCATTCCAAC
*Fabp4*	NM_024406.4	AAGGTGAAGAGCATCATAACCCT	TCACGCCTTTCATAACACATTCC
*β-actin*	NM_007393.5	GCAGGAGTACGATGAGTCCG	ACGCAGCTCAGTAACAGTCC
*Catalase*	NM_009804.2	CACACCTACACGCAGGCCGG	CTGCGCTCCGGAGTGGGAGA
*Gpx1*	NM_008160.6	AGTCCACCGTGTATGCCTTC	GAGAAGCGACATTCAATG
*Sod1*	NM_011434.2	GTGTGCGTGCTGAAGGGCGA	GACGTGGAACCCATGCTGGCC
*Sod2*	NM_013671.3	GTGGTGGAGAACCCAAAGGA	AACCTTGGACTCCCACAGACA

## Data Availability

The data are contained within the article and Supplementary Materials.

## Supplementary Materials

The following supporting information can be downloaded at: https://www.mdpi.com/2076-3921/12/3/640/s1, Figure S1: The expression profile of MT3 during different stages of 3T3-L1 adipocyte differentiation; Figure S2: *Mt3* overexpression reduces ROS scavenging gene expression in 3T3-L1 cells; Figure S3: The inhibitory effect of MT3 on adipocyte differentiation is partially reversed by antimycin A.

## References

[1] Havas S., Aronne L., Woodworth K. (2009). The problem of obesity in the United States has become increasingly prominent and is now recognized as a critical target for public health intervention. Introduction. Am. J. Med..

[2] Lindemulder S.J., Stork L.C., Lu X., Hunger S., Neglia J.P., Kadan-Lottick N.S. (2015). Reply to: Obesity is an important health problem in survivors of pediatric acute lymphoblastic leukemia. Pediatr. Blood Cancer.

[3] Williams E.P., Mesidor M., Winters K., Dubbert P.M., Wyatt S.B. (2015). Overweight and Obesity: Prevalence, Consequences, and Causes of a Growing Public Health Problem. Curr. Obes. Rep..

[4] NCD Risk Factor Collaboration (NCD-RisC) (2016). Trends in adult body-mass index in 200 countries from 1975 to 2014: A pooled analysis of 1698 population-based measurement studies with 19·2 million participants. Lancet.

[5] Azvolinsky A. (2016). The Obesity-Cancer Link: A Growing Connection. J. Natl. Cancer Inst..

[6] Argolo D.F., Iyengar N.M., Hudis C.A. (2015). Obesity and Cancer: Concepts and Challenges. Indian J. Surg. Oncol..

[7] Deng T., Lyon C.J., Bergin S., Caligiuri M.A., Hsueh W.A. (2016). Obesity, Inflammation, and Cancer. Annu. Rev. Pathol..

[8] Kusminski C.M., McTernan P.G., Kumar S. (2005). Role of resistin in obesity, insulin resistance and Type II diabetes. Clin. Sci..

[9] (2000). Obesity related to periodontal disease. J. Am. Dent. Assoc..

[10] Koutnikova H., Auwerx J. (2001). Regulation of adipocyte differentiation. Ann. Med..

[11] Cowherd R.M., Lyle R.E., McGehee R.E. (1999). Molecular regulation of adipocyte differentiation. Semin. Cell Dev. Biol..

[12] Tanaka T., Yoshida N., Kishimoto T., Akira S. (1997). Defective adipocyte differentiation in mice lacking the C/EBPbeta and/or C/EBPdelta gene. EMBO J..

[13] Tang Q.Q., Otto T.C., Lane M.D. (2003). CCAAT/enhancer-binding protein beta is required for mitotic clonal expansion during adipogenesis. Proc. Natl. Acad. Sci. USA.

[14] Park A., Kim W.K., Bae K.H. (2014). Distinction of white, beige and brown adipocytes derived from mesenchymal stem cells. World J. Stem Cells.

[15] Tontonoz P., Hu E., Spiegelman B.M. (1994). Stimulation of adipogenesis in fibroblasts by PPAR gamma 2, a lipid-activated transcription factor. Cell.

[16] Tontonoz P., Hu E., Graves R.A., Budavari A.I., Spiegelman B.M. (1994). mPPAR gamma 2: Tissue-specific regulator of an adipocyte enhancer. Genes Dev..

[17] Wu Z., Rosen E.D., Brun R., Hauser S., Adelmant G., Troy A.E., McKeon C., Darlington G.J., Spiegelman B.M. (1999). Cross-regulation of C/EBP alpha and PPAR gamma controls the transcriptional pathway of adipogenesis and insulin sensitivity. Mol. Cell.

[18] Lefterova M.I., Zhang Y., Steger D.J., Schupp M., Schug J., Cristancho A., Feng D., Zhuo D., Stoeckert C.J., Liu X.S. (2008). PPARgamma and C/EBP factors orchestrate adipocyte biology via adjacent binding on a genome-wide scale. Genes Dev..

[19] Coyle P., Philcox J.C., Carey L.C., Rofe A.M. (2002). Metallothionein: The multipurpose protein. Cell Mol. Life Sci..

[20] Albrecht A.L., Singh R.K., Somji S., Sens M.A., Sens D.A., Garrett S.H. (2008). Basal and metal-induced expression of metallothionein isoform 1 and 2 genes in the RWPE-1 human prostate epithelial cell line. J. Appl. Toxicol..

[21] Moleirinho A., Carneiro J., Matthiesen R., Silva R.M., Amorim A., Azevedo L. (2011). Gains, losses and changes of function after gene duplication: Study of the metallothionein family. PLoS ONE.

[22] Vasák M., Hasler D.W. (2000). Metallothioneins: New functional and structural insights. Curr. Opin. Chem. Biol..

[23] Palmiter R.D. (1998). The elusive function of metallothioneins. Proc. Natl. Acad. Sci. USA.

[24] Maret W. (2000). The function of zinc metallothionein: A link between cellular zinc and redox state. J. Nutr..

[25] Sharma S., Moon C.S., Khogali A., Haidous A., Chabenne A., Ojo C., Jelebinkov M., Kurdi Y., Ebadi M. (2013). Biomarkers in Parkinson’s disease (recent update). Neurochem. Int..

[26] Klaassen C.D., Liu J., Diwan B.A. (2009). Metallothionein protection of cadmium toxicity. Toxicol. Appl. Pharmacol..

[27] Si M., Lang J. (2018). The roles of metallothioneins in carcinogenesis. J. Hematol. Oncol..

[28] Adam P., Křížková S., Heger Z., Babula P., Pekařík V., Vaculovičoá M., Gomes C.M., Kizek R., Adam V. (2016). Metallothioneins in Prion- and Amyloid-Related Diseases. J. Alzheimers Dis..

[29] Beattie J.H., Wood A.M., Newman A.M., Bremner I., Choo K.H., Michalska A.E., Duncan J.S., Trayhurn P. (1998). Obesity and hyperleptinemia in metallothionein (-I and -II) null mice. Proc. Natl. Acad. Sci. USA.

[30] Sato M., Kawakami T., Kondoh M., Takiguchi M., Kadota Y., Himeno S., Suzuki S. (2010). Development of high-fat-diet-induced obesity in female metallothionein-null mice. FASEB J..

[31] Byun H.R., Kim D.K., Koh J.Y. (2011). Obesity and downregulated hypothalamic leptin receptors in male metallothionein-3-null mice. Neurobiol. Dis..

[32] Sena L.A., Chandel N.S. (2012). Physiological roles of mitochondrial reactive oxygen species. Mol. Cell.

[33] Murphy M.P., Holmgren A., Larsson N.G., Halliwell B., Chang C.J., Kalyanaraman B., Rhee S.G., Thornalley P.J., Partridge L., Gems D. (2011). Unraveling the biological roles of reactive oxygen species. Cell. Metab..

[34] Imhoff B.R., Hansen J.M. (2010). Extracellular redox environments regulate adipocyte differentiation. Differentiation.

[35] Wilson-Fritch L., Burkart A., Bell G., Mendelson K., Leszyk J., Nicoloro S., Czech M., Corvera S. (2003). Mitochondrial biogenesis and remodeling during adipogenesis and in response to the insulin sensitizer rosiglitazone. Mol. Cell Biol..

[36] Sekiya M., Hiraishi A., Touyama M., Sakamoto K. (2008). Oxidative stress induced lipid accumulation via SREBP1c activation in HepG2 cells. Biochem. Biophys. Res. Commun..

[37] Lee H., Lee Y.J., Choi H., Ko E.H., Kim J.W. (2009). Reactive oxygen species facilitate adipocyte differentiation by accelerating mitotic clonal expansion. J. Biol. Chem..

[38] Ruttkay-Nedecky B., Nejdl L., Gumulec J., Zitka O., Masarik M., Eckschlager T., Stiborova M., Adam V., Kizek R. (2013). The role of metallothionein in oxidative stress. Int. J. Mol. Sci..

[39] Han Y., Lee S.H., Bahn M., Yeo C.Y., Lee K.Y. (2016). Pin1 enhances adipocyte differentiation by positively regulating the transcriptional activity of PPARγ. Mol. Cell Endocrinol..

[40] Li S., Kim M.J., Lee S.H., Jin L., Cong W., Jeong H.G., Lee K.Y. (2021). Metallothionein 3 Promotes Osteoblast Differentiation in C2C12 Cells via Reduction of Oxidative Stress. Int. J. Mol. Sci..

[41] Chen Q., Shou P., Zheng C., Jiang M., Cao G., Yang Q., Cao J., Xie N., Velletri T., Zhang X. (2016). Fate decision of mesenchymal stem cells: Adipocytes or osteoblasts?. Cell Death Differ..

[42] Berendsen A.D., Olsen B.R. (2014). Osteoblast-adipocyte lineage plasticity in tissue development, maintenance and pathology. Cell Mol. Life Sci..

[43] Hu L., Yin C., Zhao F., Ali A., Ma J., Qian A. (2018). Mesenchymal Stem Cells: Cell Fate Decision to Osteoblast or Adipocyte and Application in Osteoporosis Treatment. Int. J. Mol. Sci..

[44] Achari A.E., Jain S.K. (2017). Adiponectin, a Therapeutic Target for Obesity, Diabetes, and Endothelial Dysfunction. Int. J. Mol. Sci..

[45] Jackson R.M., Griesel B.A., Gurley J.M., Szweda L.I., Olson A.L. (2017). Glucose availability controls adipogenesis in mouse 3T3-L1 adipocytes via up-regulation of nicotinamide metabolism. J. Biol. Chem..

[46] Berndt J., Kovacs P., Ruschke K., Klöting N., Fasshauer M., Schön M.R., Körner A., Stumvoll M., Blüher M. (2007). Fatty acid synthase gene expression in human adipose tissue: Association with obesity and type 2 diabetes. Diabetologia.

[47] Han Y.H., Kim S.H., Kim S.Z., Park W.H. (2008). Antimycin A as a mitochondria damage agent induces an S phase arrest of the cell cycle in HeLa cells. Life Sci..

[48] Trayhurn P., Duncan J.S., Wood A.M., Beattie J.H. (2000). Metallothionein gene expression and secretion in white adipose tissue. Am. J. Physiol. Regul. Integr. Comp. Physiol..

[49] Cancello R., Zulian A., Gentilini D., Mencarelli M., Della Barba A., Maffei M., Vitti P., Invitti C., Liuzzi A., Di Blasio A.M. (2013). Permanence of molecular features of obesity in subcutaneous adipose tissue of ex-obese subjects. Int. J. Obes..

[50] Haynes V., Connor T., Tchernof A., Vidal H., Dubois S. (2013). Metallothionein 2a gene expression is increased in subcutaneous adipose tissue of type 2 diabetic patients. Mol. Genet. Metab..

[51] Kawakami T., Takasaki S., Kadota Y., Fukuoka D., Sato M., Suzuki S. (2019). Regulatory role of metallothionein-1/2 on development of sex differences in a high-fat diet-induced obesity. Life Sci..

[52] Lindeque J.Z., Jansen van Rensburg P.J., Louw R., van der Westhuizen F.H., Florit S., Ramírez L., Giralt M., Hidalgo J. (2015). Obesity and metabolomics: Metallothioneins protect against high-fat diet-induced consequences in metallothionein knockout mice. OMICS.

[53] Kadota Y., Toriuchi Y., Aki Y., Mizuno Y., Kawakami T., Nakaya T., Sato M., Suzuki S. (2017). Metallothioneins regulate the adipogenic differentiation of 3T3-L1 cells via the insulin signaling pathway. PLoS ONE.

[54] Sarjeant K., Stephens J.M. (2012). Adipogenesis. Cold Spring Harb. Perspect. Biol..

[55] Howells C., West A.K., Chung R.S. (2010). Neuronal growth-inhibitory factor (metallothionein-3): Evaluation of the biological function of growth-inhibitory factor in the injured and neurodegenerative brain. FEBS J..

[56] Uchida Y., Gomi F., Masumizu T., Miura Y. (2002). Growth inhibitory factor prevents neurite extension and the death of cortical neurons caused by high oxygen exposure through hydroxyl radical scavenging. J. Biol. Chem..

[57] You H.J., Lee K.J., Jeong H.G. (2002). Overexpression of human metallothionein-III prevents hydrogen peroxide-induced oxidative stress in human fibroblasts. FEBS Lett..

[58] Kanda Y., Hinata T., Kang S.W., Watanabe Y. (2011). Reactive oxygen species mediate adipocyte differentiation in mesenchymal stem cells. Life Sci..

[59] Tormos K.V., Anso E., Hamanaka R.B., Eisenbart J., Joseph J., Kalyanaraman B., Chandel N.S. (2011). Mitochondrial complex III ROS regulate adipocyte differentiation. Cell Metab..

[60] Kim J.H., Kim S.H., Song S.Y., Kim W.S., Song S.U., Yi T., Jeon M.S., Chung H.M., Xia Y., Sung J.H. (2014). Hypoxia induces adipocyte differentiation of adipose-derived stem cells by triggering reactive oxygen species generation. Cell Biol. Int..

[61] Lee K.S., Kim S.R., Park S.J., Park H.S., Min K.H., Jin S.M., Lee M.K., Kim U.H., Lee Y.C. (2006). Peroxisome proliferator activated receptor-gamma modulates reactive oxygen species generation and activation of nuclear factor-kappaB and hypoxia-inducible factor 1alpha in allergic airway disease of mice. J. Allergy Clin. Immunol..

[62] Chen K., Chen J., Li D., Zhang X., Mehta J.L. (2004). Angiotensin II regulation of collagen type I expression in cardiac fibroblasts: Modulation by PPAR-gamma ligand pioglitazone. Hypertension.

[63] Srivastava N., Kollipara R.K., Singh D.K., Sudderth J., Hu Z., Nguyen H., Wang S., Humphries C.G., Carstens R., Huffman K.E. (2014). Inhibition of cancer cell proliferation by PPARγ is mediated by a metabolic switch that increases reactive oxygen species levels. Cell Metab..

[64] Bost F., Aouadi M., Caron L., Binetruy B. (2005). The role of MAPKs in adipocyte differentiation and obesity. Biochimie.

[65] Pearson G., Robinson F., Beers Gibson T., Xu B.E., Karandikar M., Berman K., Cobb M.H. (2001). Mitogen-activated protein (MAP) kinase pathways: Regulation and physiological functions. Endocr. Rev..

[66] Tang Q.Q., Otto T.C., Lane M.D. (2003). Mitotic clonal expansion: A synchronous process required for adipogenesis. Proc. Natl. Acad. Sci. USA.

[67] Hu E., Kim J.B., Sarraf P., Spiegelman B.M. (1996). Inhibition of adipogenesis through MAP kinase-mediated phosphorylation of PPARgamma. Science.

[68] Kusuyama J., Ohnishi T., Bandow K., Amir M.S., Shima K., Semba I., Matsuguchi T. (2017). Constitutive activation of p46JNK2 is indispensable for C/EBPdelta induction in the initial stage of adipogenic differentiation. Biochem. J..

[69] Engelman J.A., Lisanti M.P., Scherer P.E. (1998). Specific inhibitors of p38 mitogen-activated protein kinase block 3T3-L1 adipogenesis. J. Biol. Chem..

[70] Cho R.L., Yang C.C., Lee I.T., Lin C.C., Chi P.L., Hsiao L.D., Yang C.M. (2016). Lipopolysaccharide induces ICAM-1 expression via a c-Src/NADPH oxidase/ROS-dependent NF-kappaB pathway in human pulmonary alveolar epithelial cells. Am. J. Physiol. Lung. Cell Mol. Physiol..

[71] Kuo C.W., Shen C.J., Tung Y.T., Chen H.L., Chen Y.H., Chang W.H., Cheng K.C., Yang S.H., Chen C.M. (2015). Extracellular superoxide dismutase ameliorates streptozotocin-induced rat diabetic nephropathy via inhibiting the ROS/ERK1/2 signaling. Life Sci..

[72] McCubrey J.A., Lahair M.M., Franklin R.A. (2006). Reactive oxygen species-induced activation of the MAP kinase signaling pathways. Antioxid. Redox Signal..

[73] Son Y., Cheong Y.K., Kim N.H., Chung H.T., Kang D.G., Pae H.O. (2011). Mitogen-Activated Protein Kinases and Reactive Oxygen Species: How Can ROS Activate MAPK Pathways?. J. Signal. Transduct..

[74] Sakaue H., Ogawa W., Matsumoto M., Kuroda S., Takata M., Sugimoto T., Spiegelman B.M., Kasuga M. (1998). Posttranscriptional control of adipocyte differentiation through activation of phosphoinositide 3-kinase. J. Biol. Chem..

[75] Yun S.J., Kim E.K., Tucker D.F., Kim C.D., Birnbaum M.J., Bae S.S. (2008). Isoform-specific regulation of adipocyte differentiation by Akt/protein kinase Balpha. Biochem. Biophys. Res. Commun..

[76] Martindale J.L., Holbrook N.J. (2002). Cellular response to oxidative stress: Signaling for suicide and survival. J. Cell. Physiol..

[77] Annie-Mathew A.S., Prem-Santhosh S., Jayasuriya R., Ganesh G., Ramkumar K.M., Sarada D.V.L. (2021). The pivotal role of Nrf2 activators in adipocyte biology. Pharmacol. Res..

