# Supplementary material for: Metallothionein 3 Inhibits 3T3-L1 Adipocyte Differentiation via Reduction of Reactive Oxygen Species
Source: Antioxidants (Basel). 2023 Mar 4;12(3):640. doi: 10.3390/antiox12030640 (PMC10045306; doi:10.3390/antiox12030640)
Supplement: Supplementary file 1 [file antioxidants-12-00640-s001.zip › antioxidants-2143521-supplementary.pdf]

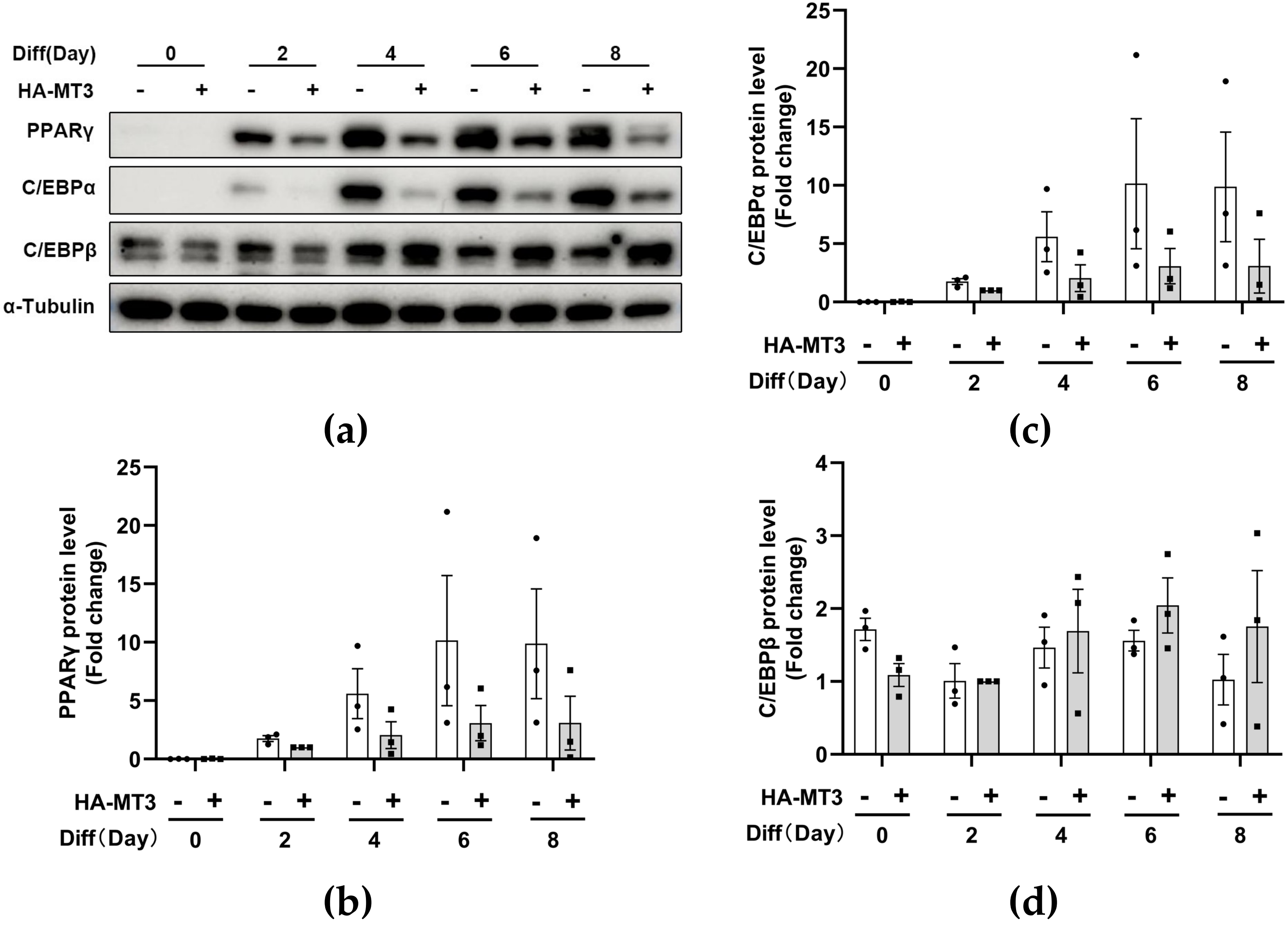

Figure S1. The expression profile of MT3 during different stages of 3T3-L1 adipocyte differentiation. The differentiation of 3T3-L1 adipocyte was induced by the differentiation cocktail. The protein samples were harvested at specified times (day 0, 2, 4, 6, and 8). (a) Immunoblotting was used to detect the protein levels of PPARγ, C/EBPα, C/EBPβ, and α-Tubulin. α-Tubulin was used as a loading control. (b–d) The intensities of bands (PPARγ, C/EBPα, C/EBPβ) were quantified and normalized with the corresponding α-Tubulin bands. The data are presented as the means ± SEM.

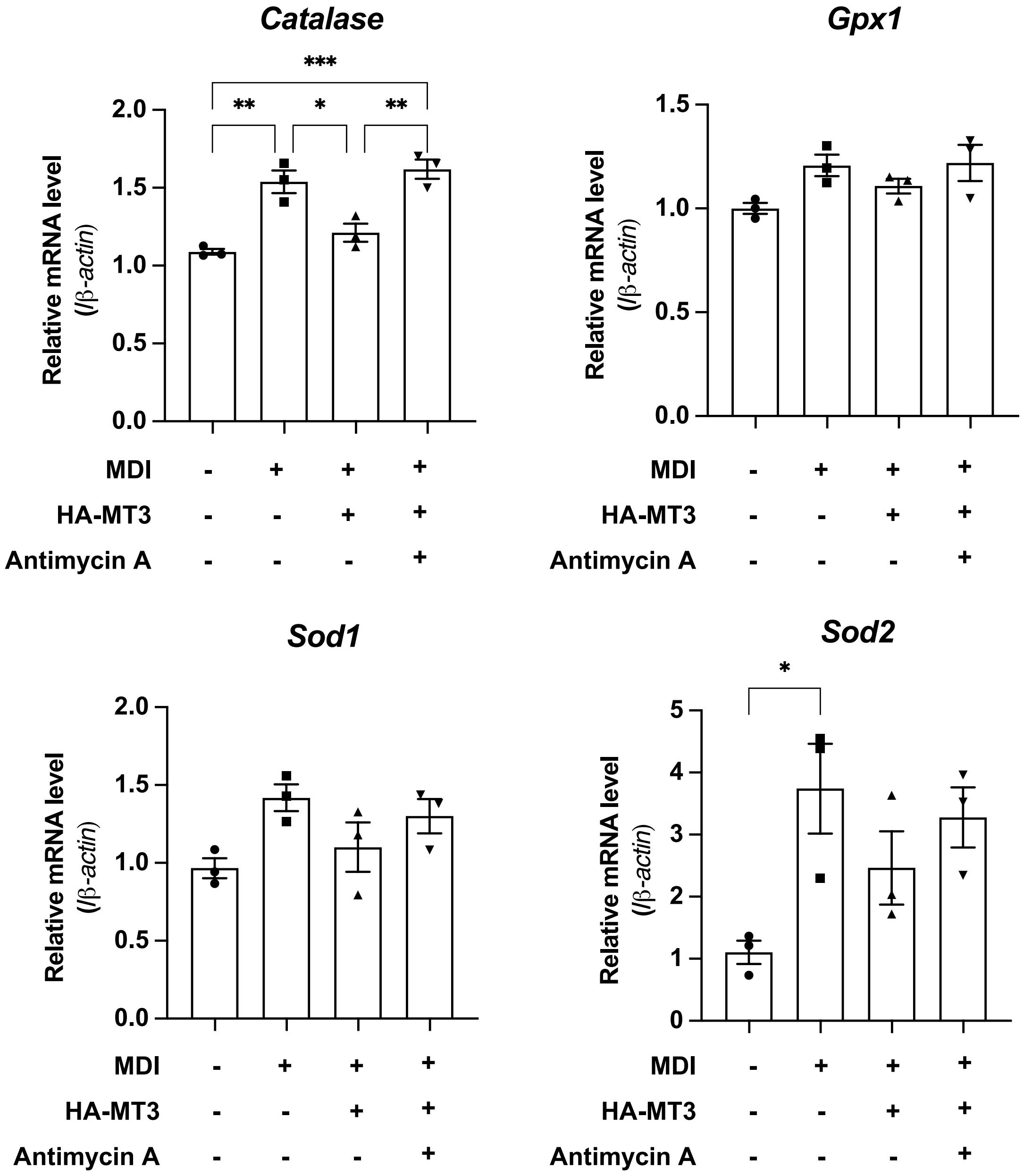

Figure S2. *Mt3* overexpression reduces ROS scavenging gene expression in 3T3-L1 cells. 3T3-L1 cells transfected with HA-MT3 plasmid were differentiated induced by differentiation cocktail and treated with or without 100 nM antimycin A for 12 h. The mRNA levels of ROS scavenging genes *Catalase*, *Gpx1*, *Sod1, and Sod2* in the different groups were compared by RT-qPCR. *β-actin* was used as a loading control. The data are presented as the means ± SEM. *P < 0.05, **P < 0.01, and ***P < 0.001were calculated by using one-way ANOVA, followed by a Tukey's Test.

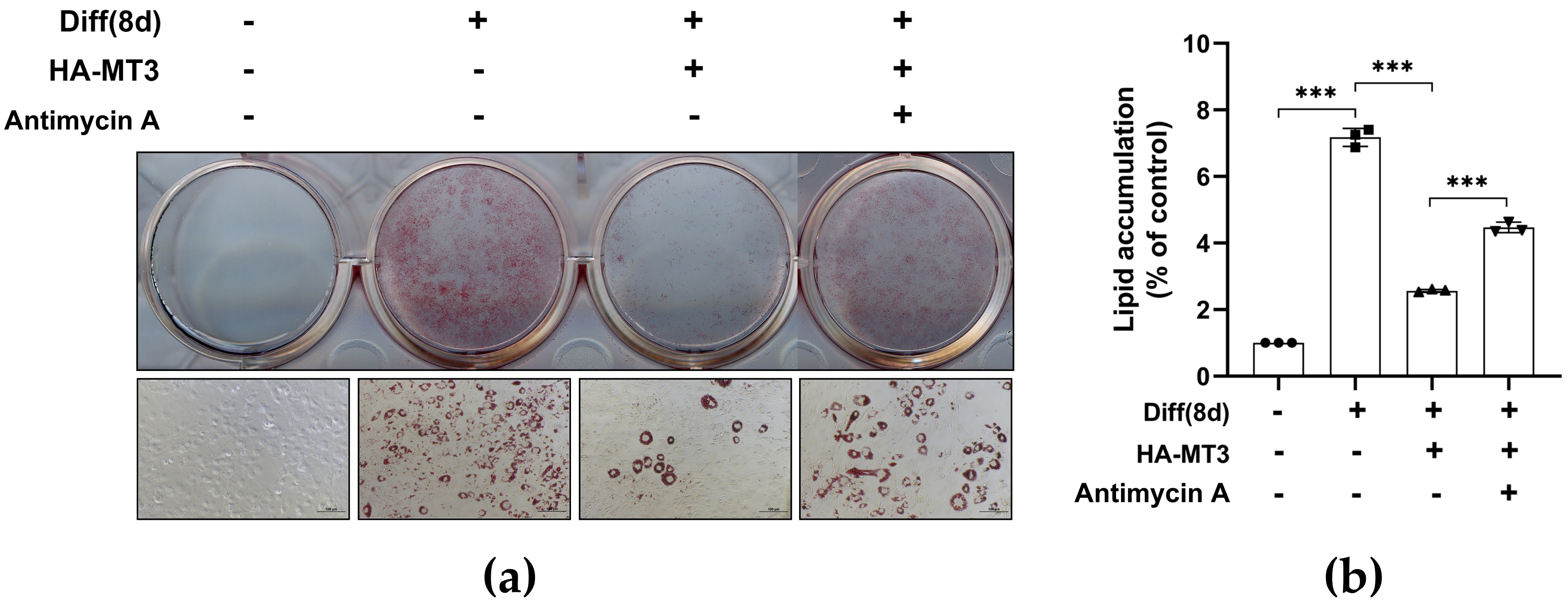

Figure S3. The inhibitory effect of MT3 on adipocyte differentiation is partially reversed by antimycin A. 3T3-L1 cells transfected with or without HA-MT3 plasmid were incubated with differentiation cocktail to induce adipocyte differentiation and treated with or without 25 nM antimycin A for 8 days. (a) Representative images of 3T3-L1 adipocytes were captured at 200× magnification. Scale bar = 100 μm. (b) The lipid accumulation in 3T3-L1 adipocytes was quantified as a percentage of control values. ***$P < 0.001$ vs. the corresponding undifferentiated group & differentiated group were calculated by using one-way ANOVA, followed by a Tukey's Test.
